# Myxoid liposarcoma of sigmoid mesocolon: a case report and review of the literature

**DOI:** 10.1186/s13256-026-05915-1

**Published:** 2026-03-04

**Authors:** Raed R. Hamed, Abbas W. Abbas, Lila H. Abu-Hilal, Ayah Abulehia, Omar H. Abu Zaydeh, Tawfiq Abukeshek, Izzedin A. Bakri, Bashar Jaber

**Affiliations:** 1 Department of General Surgery, Al-Makassed Islamic Charitable Hospital, East Jerusalem, Palestine; 2 Faculty of Medicine, Al-Quds University, East Jerusalem, Palestine; 3 Department of Radiology, Al-Makassed Islamic Charitable Hospital, East Jerusalem, Palestine; 4 Department of Histopathology, Al-Makassed Islamic Charitable Hospital, East Jerusalem, Palestine

**Keywords:** Myxoid liposarcoma, Mesocolon, Resection, Adjuvant radiotherapy, Case report

## Abstract

**Background:**

Liposarcoma is a malignant mesenchymal tumor most commonly arising in the extremities and retroperitoneum. Involvement of the mesentery is rare, and primary liposarcoma of the large bowel mesentery has been reported fewer than 15 times in the English literature. Among its histological subtypes, myxoid liposarcoma is exceptionally rare in this location, with only four previous cases described. Owing to its rarity, there are no standardized management protocols, although wide surgical excision with negative margins remains the mainstay of treatment.

**Case presentation:**

We report a 73 year-old Arab male with hypertension and ischemic heart disease who presented with painless abdominal swelling and anorexia. Imaging revealed a large left mesocolon mass, and a left hemicolectomy with colosigmoid anastomosis achieved R0 margins. Histopathology confirmed primary myxoid liposarcoma of the mesocolon. The patient had an uneventful recovery and remains disease-free 1 year after surgery.

**Conclusion:**

Primary myxoid liposarcoma of the mesocolon is extremely rare, with this case representing only the fifth reported instance. Review of the literature indicates that complete surgical resection is the cornerstone of management, while the role of adjuvant radiotherapy or chemotherapy remains controversial. Reporting additional cases is essential to improve understanding, refine prognostic assessment, and guide the development of evidence-based treatment strategies.

## Background

Liposarcoma is a malignant mesenchymal tumor arising from adipose tissue, most commonly located in the extremities and retroperitoneum, though rare sites such as the small and large bowel mesentery can also be affected [1, 2]. According to the World Health Organization (WHO), liposarcomas are classified into five histologic subtypes with variable clinical behavior and prognosis [1]. Myxoid liposarcoma is an uncommon variant characterized by distinctive histopathological and radiological features. Primary involvement of the mesocolon is extremely rare, with only four cases previously reported in the English literature [2].

The optimal management of mesocolon liposarcoma is not well established because of its rarity, but complete surgical resection with negative margins is generally regarded as the treatment of choice. The role of adjuvant radiotherapy or chemotherapy remains uncertain and is usually considered in high-risk cases [1–6]. This report adds a further case of primary myxoid liposarcoma of the mesocolon and reviews the available literature to improve understanding of this rare disease.

## Case presentation

A 73-year-old Palestinian Arab male patient, with a past medical history of hypertension, ischemic heart disease (IHD), and heart failure, and no family history of malignancy, presented with a sensation of painless abdominal swelling over the left side of the abdomen, which started 2 weeks prior to presentation. There was a history of anorexia without significant weight loss. The patient reported dark colored stool without bright red blood. Otherwise, he did not have any other complaints.

Clinical examination revealed pallor, while abdominal examination showed distention due to a hard, nontender irregular lobulated mass occupying the left side of the abdomen, measuring about 20 × 15 cm. Digital rectal examination (DRE) was unremarkable. Laboratory investigations showed anemia with a hemoglobin (Hb) level of 10.8 g/dL. He also had elevated kidney function tests, attributed first to acute kidney injury (AKI) due to previous intravenous contrast taken a few days prior to presentation. The fecal occult blood test was negative. Tumor markers, including carcinoembryonic antigen (CEA) and cancer antigen 19.9 (CA 19.9), were within normal range.

**Fig. 1:**
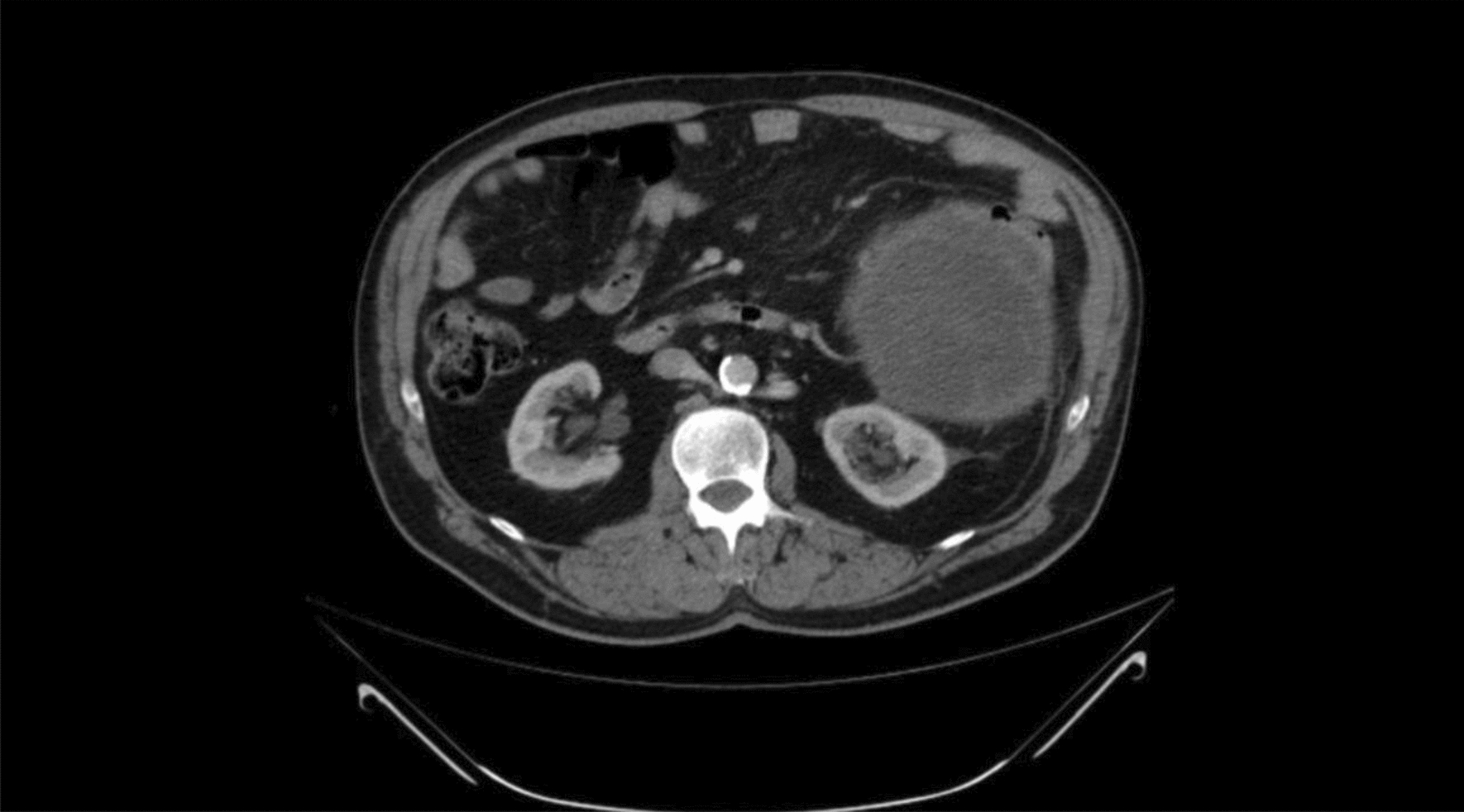
Axial abdominal computed tomography postcontrast study (portal phase) showing large, well-defined lesion with mass effect over the the lower descending colon, pushing it

**Fig. 2:**
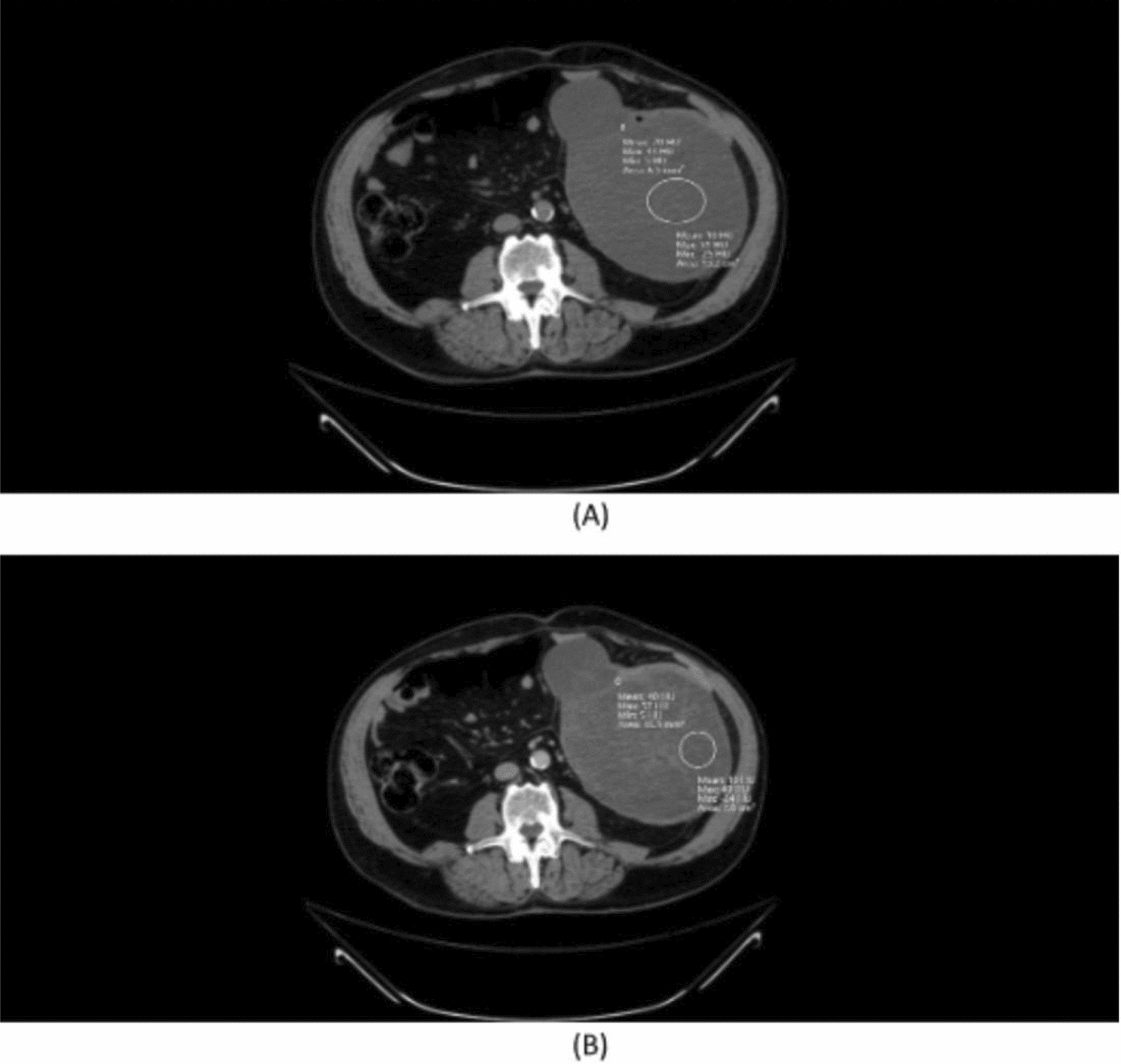
Axial abdominal computed tomography precontrast (**A**) and post contrast (**B**) showing density stability of the cystic portion (large circle with mean density of 10 Hounsfield units on both images)

A chest, abdomen, and pelvic computed tomography (CT) scan revealed a large soft tissue mass occupying the left abdomen, measuring 17 × 15 × 13 cm, with a mass effect pushing the lower descending colon anteriorly (Fig. 1). The lesion was well-defined and predominantly hypodense with a cystic portion and fine internal septations. The predominant hypodense portion had a water density of 10 Hounsfield units (HU) on the precontrast study and remained stable on the post-contrast study (portal phase) with no enhancement. However, the septations, which had soft tissue density of 20 HU on the precontrast study, showed significant enhancement on the postcontrast study, measuring 40 HU (+ 20 HU) (Fig. 2). There were no calcifications or fat components, and no convincing site of origin could be identified; however, it appeared to be arising from the large bowel mesentery. No focal liver lesions, lung lesions, or any other significant pathology were noted. The differential diagnosis included gastrointestinal stromal tumor (GIST) or desmoid tumor.

Upper endoscopy revealed a small sessile gastric polyp at the fundus, which was identified as a hyperplastic polyp negative for dysplasia and malignancy upon polypectomy.

Colonoscopy showed a 10-cm polypoid tumor in the proximal descending colon, with the rest of the colon appearing normal. Multiple biopsies taken from the mass showed necrotic tissue without evidence of malignancy. The patient did not receive any neoadjuvant therapy.

**Fig. 3:**
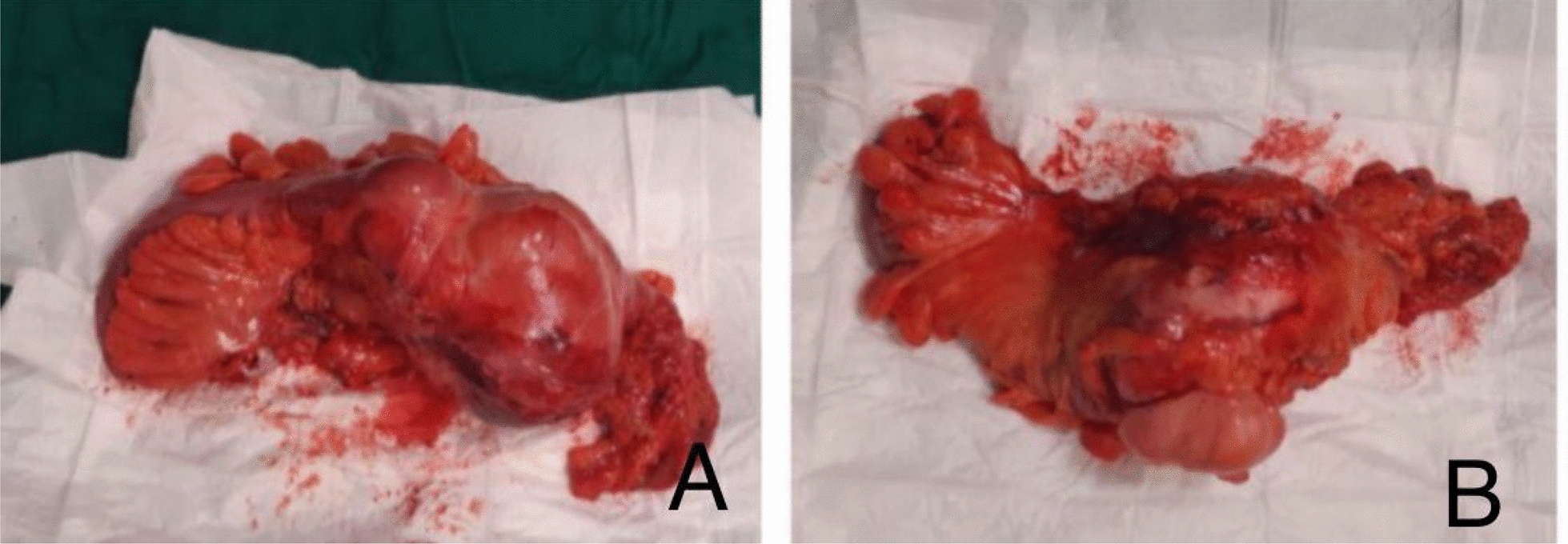
The resected specimen showing the left colon mass arising from the mesocolon and invading the wall of the colon. **A** Anterior view; **B** Posterior/lateral view

After proper patient preparation and normalization of kidney function tests, exploratory laparotomy was performed. Intraoperative findings revealed a huge descending colon tumor occupying the left abdomen, compressing the left ureter and kidney. Consequently, left hemicolectomy with side-to-side primary colosigmoid anastomosis was performed, with preservation of the ureter and kidney (Fig. 3). The patient’s acute kidney injury on initial presentation was likely multifactorial, primarily related to recent intravenous contrast administration prior to admission to our hospital, with a possible contribution from ureteral compression by the tumor, as confirmed intraoperatively, but unfortunately there was no prior abdominal imaging report available for comparison. The postoperative course was uneventful, and the patient was discharged home a few days later on a regular diet after the return of usual bowel function.

**Fig. 4:**
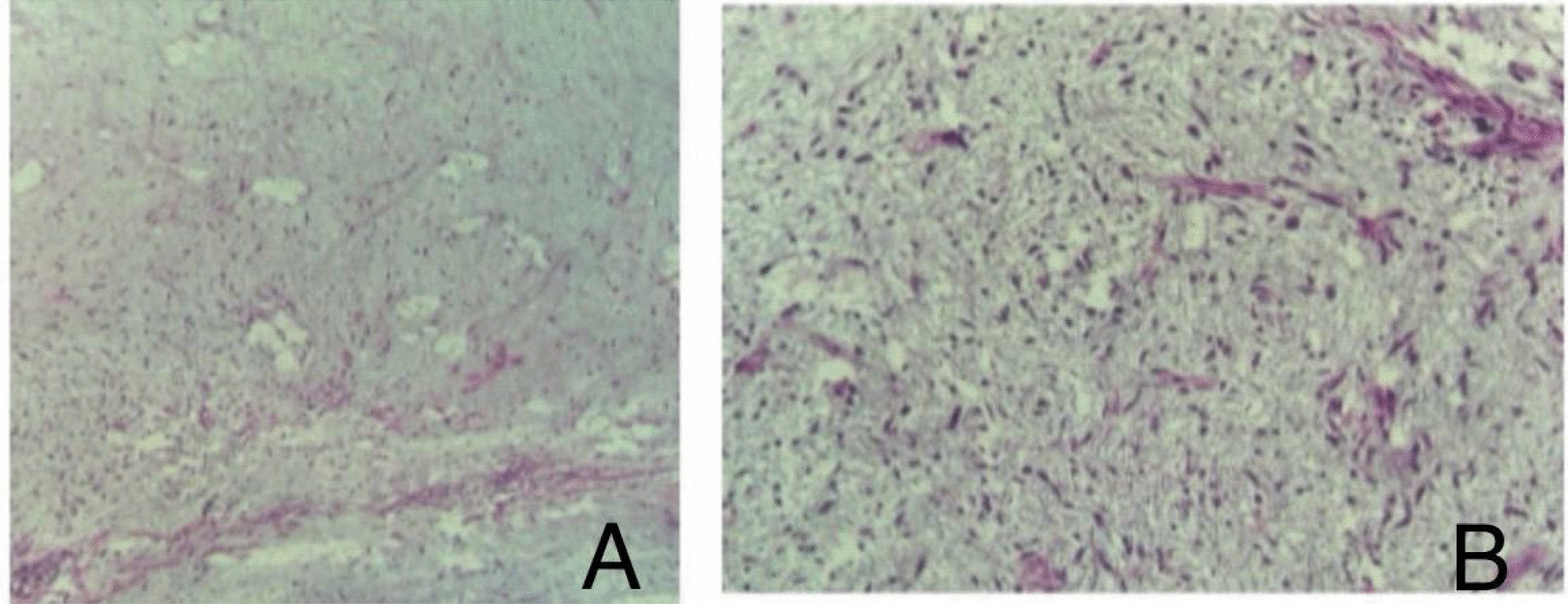
Histopathological examination of the resected mesocolon mass showing features of myxoid liposarcoma. Hematoxylin–eosin staining at (**A**) 10 × and (**B**) 40 × magnification

Histopathological examination of the specimen showed a myxoid soft tissue neoplasm consistent with mesocolon myxoid liposarcoma (Fig. 4). The resection margins were free of tumor, and there was no lymphovascular invasion. The examined retrieved lymph nodes were negative for malignancy. The tumor’s pathologic stage was defined as PT3, N0, M0. Ancillary studies showed weakly positive S100, while Desmin, epithelial membrane antigen (EMA), B-Catenin, and cluster of differentiation-99 (CD-99) were all negative.

Postsurgery, the oncology team opted for follow-up without adjuvant treatment. After 1 year, no disease recurrence has been documented. The clinical course and key events are summarized in the timeline (Table 1).

**Table 1:** Timeline table

Patient presentation (day 0)	73-year-old male with hypertension, ischemic heart disease, and heart failure presents with painless left-sided abdominal swelling lasting 2 weeks. Reports anorexia and melena. No significant weight loss. No family history of malignancy
Clinical examination (day 0)	Pallor noted. Abdominal exam reveals a 20 × 15 cm irregular, nontender mass on the left side. Digital rectal examination unremarkable
Laboratory investigations (day 0)	Anemia (Hb = 10.8 g/dL). Elevated kidney function tests (AKI due to prior contrast). Negative fecal occult blood test. Normal tumor markers (CEA, CA 19.9)
Imaging (day 0)	CT scan shows a 17 × 15 × 13 cm soft tissue mass in the left abdomen, appearing to originate from the large bowel mesentery. No liver or lung lesions noted. Differential diagnosis: GIST or desmoid tumor
Upper endoscopy (day 3)	A small sessile gastric polyp identified, found to be hyperplastic, negative for dysplasia/malignancy
Colonoscopy (day 3)	10-cm polypoid tumor in the proximal descending colon, biopsies show necrotic tissue, no malignancy
Presurgery (day 4–7)	Patient prepared, kidney function normalized. No neoadjuvant therapy
Exploratory laparotomy (day 1)	Intraoperative findings: large descending colon tumor compressing left kidney and ureter. Left hemicolectomy and colosigmoid anastomosis performed
Postoperative (day 1–2)	Uneventful recovery, discharged on a regular diet after bowel function returned
Histopathology (day 14 postoperative)	Myxoid liposarcoma of the mesocolon confirmed. Free resection margins, no lymphovascular invasion. Lymph nodes negative for malignancy. Pathologic stage: PT3, N0, M0
Ancillary studies (day 14)	Weakly positive for S100. Negative for Desmin, EMA, B-Catenin, CD-99
Postsurgery follow-up (1 year)	No disease recurrence after 1 year. Follow-up without adjuvant treatment

## Discussion

Liposarcoma is a common malignant tumor that arises from the mesenchyme of adipose tissue. The most common locations for development of liposarcoma are the extremities and retroperitoneum; however, it can also affect other extremely rare sites such as the mesentery of small and large bowels [1, 2]. Up to 2019, only 10 cases were described in the English literature about the primary mesenteric liposarcoma of the colon [2]. Since then, the number has not changed much. Therefore, it is very important to have more studies related to the disease for optimal management.

Males are slightly more likely to be affected by primary mesenteric liposarcoma compared with females. The incidence of the disease is higher in the 5th to 7th decades [3, 4]. However, it can present in any age group and was diagnosed in patients as young as 15 years old [1].

Soft tissue liposarcoma is classified into five types according to the World Health Organization (WHO) classification [1]. Each of the types has different incidence, recurrence rate, prognosis, and mortality rate. The atypical lipomatous tumor “ALT”/well differentiated “WD” is the most common liposarcoma, accounting for about 40–45% of the cases. The recurrence rate approaches zero when the tumor is completely surgically removed with clear margins. The prognosis of WD tumors depends on the depth of the tumors, and the mortality rate depends on the site of the tumor; 0% in extremities while it can reach up to 80% in retroperitoneum. The survival rate is usually 6–11 years when followed up for 10–20 years [1–3].

Dedifferentiated and myxoid liposarcoma has the same incidence, approaching 10% for each of them. The dedifferentiated type has about 40% local recurrence and 15–20% for distant metastasis. It has a mortality rate of about 30% within a 5-year follow-up. Retroperitoneum carries the worst prognosis compared with other sites of the same tumor [4].

Regarding myxoid liposarcoma, it is a malignancy containing uniform round to oval primitive nonlipogenic mesenchymal cells and a number of small signet-ring lipoblasts in a myxoid stroma with a characteristic branching vascular pattern [2]. The lower extremities are more commonly affected by this type of tumor, comprising about two-thirds of the cases. It is more prone to local recurrence, and one-third of the patients may develop distant metastasis [1]. The sites of possible metastasis include cardiac, hepatic, mesenteric, bone, and pulmonary [2]. Certain factors play a role in unfavorable prognosis, such as a high histological grade (> 5% RC areas), presence of necrosis, and TP53 overexpression [1]. The survival rate ranges between 6 and 20 years [2].

Pleomorphic liposarcoma is an extremely rare tumor with a high recurrence and mortality rate, both approaching 50% in all cases [1]. The prognosis depends on the tumor depth, size, number of mitosis in 10 high-power fields (HPFs), and presence of necrosis. The mixed type is an extremely rare type of liposarcoma.

Thus far, there are only four established cases of primary myxoid liposarcoma of the large bowel mesentery [2]. This makes the reported case in this article the fifth one in English literature.

The clinical picture of primary mesenteric liposarcoma varies significantly depending on the age, pathology, and tumor size [1, 2]. The insidious course of the tumor makes diagnosis challenging and delayed [2]. Differential diagnosis of primary mesenteric liposarcoma include lymphoma, which is the most common solid tumor in the mesentery, as well as desmoid tumor, Castleman’s disease, and metastatic lymph nodes from neuroendocrine tumors [2]. The main presenting symptoms of a patient with mesenteric liposarcoma are abdominal pain, presence of mobile abdominal mass, and weight loss [4]. Other symptoms such as vomiting and constipation could be present in cases with giant masses [1]. Although most of the reported cases have been single, multiple mesenteric liposarcomas have been described before [5]. When multicentre liposarcomas are encountered, it is very important to differentiate between secondary tumors or independently arising multicentric liposarcomas, because the latter diagnosis requires a more aggressive management [6].

CT and magnetic resonance imaging (MRI) have been valuable tools in suggesting the diagnosis and determining the size and invasion of the mass to adjacent organs [3]. The usual CT characteristics that may give a clue for mesenteric liposarcoma include heterogeneity, contrast enhancement, infiltration or poor margination, and less attenuation compared with muscle [1, 3]. The degree of enhancement corresponds to the degree of histological grade [3]. The myxoid liposarcoma has a distinctive characteristic of appearing cystic on CT attenuation before contrast enhancement and then appearing to be solid after contrast enhancement [1]. Magnetic resonance imaging (MRI) can also help in cases of mesenteric liposarcomas as their signal intensity is similar to that of water (i.e. low on T1-weighted images and high on T2-weighted images) [6]. However, myxoid type should be differentiated from benign cysts by careful inspection and contrast enhancement. Myxoid component usually shows specific findings of lacy, reticular, linear, or amorphous regions. In particular, T2-weighted images often display septa of low signal intensity within these components [6]. The use of MRI can also aid in achieving complete surgical resection by defining the reaction zone [2]. Despite the important information gained from the imaging, histopathological examination remains the gold standard for definitive diagnosis [7]. However, performing preoperative biopsy from mesenteric liposarcoma is still widely debatable due to some reasons, including the lack of adequate sample and the possible false results due to the inability to get thorough sampling from different sites of the tumor to identify the nonlipogenic component, which helps in differentiating between the well-differentiated liposarcoma from other types [7, 8]. In our case, colonoscopy revealed a polypoid tumor, but the biopsy was inconclusive for malignancy. This may be explained by the tumor’s heterogeneous composition and the difficulty in sampling adequate tissue, particularly when necrotic or myxoid areas predominate. Furthermore, the presence of necrotic areas in the tumor makes the biopsy result inconclusive.

Previous studies have agreed that the standard strategy for treating mesenteric liposarcoma has been wide surgical excision with safety margins [1–6]. Complete surgical resection has shown to be superior to any other treatment approach in terms of long-term survival and distant recurrence-free survival rates [8, 9]. Sometimes, en-bloc resection of the nearby organs that are invaded by the tumor is required to achieve negative margins (R0) [8]. The safety margins for complete resection of mesenteric liposarcoma have been described to be at least 1 cm away from the tumor or the reaction zone defined by MRI. However, owing to late presentation and the massive size of the tumor encountered at the time of presentation, accomplishment of complete resection is difficult. Positive surgical margins have been accused to be the main predictors of local relapse [9].

Histologically, myxoid liposarcomas demonstrate the morphologic and immunohistochemical features of their constituent components. At low magnification, they are moderately cellular, lobulated tumors with increased peripheral cellularity, comprising patternless arrays of uniform, small, ovoid cells without morphological adipocytic differentiation, and with variable number of small lipoblasts. The tumors contain abundant, lightly basophilic, myxoid stroma with a striking plexiform, delicately arborizing capillary network (chicken wire) around which neoplastic cells often cluster. Paucicellular extracellular mucin pools are present, imparting a microcytic pulmonary-edema like pattern. Myxoid liposarcomas typically lack atypia, substantial mitotic activity or spindling. The lipoblasts are smaller in size compared with other types of liposarcoma. The lipoblasts are predominantly univacuolated or bivacuolated. They are rare or may be absent [9].

The role of neoadjuvant therapy is still controversial and lacks sufficient studies to approve any type of treatment for mesenteric liposarcoma. Ishiguro *et al*. recommended neoadjuvant chemotherapy for large mesenteric liposarcomas [9]. The recommendation comes after reporting successful management of initially unresectable right mesocolon liposarcoma with neoadjuvant chemotherapy (doxorubicin, cisplatin, and ifosfamide). The tumor then could be resected, though R2, where postchemotherapy, the tumor demonstrated a 50% reduction of its original size. The patient then received radiotherapy and became free from recurrence for a total of 26 months. In general, the survival rates of mesenteric liposarcomas treated with neoadjuvant chemotherapy is still not well-established. However, doxorubicin remains the key chemotherapy drug while ifosfamide, dacarbazine, and cisplatin have also been used [10].

Adjuvant radiotherapy is highly recommended after surgical management for high-risk patients [1, 3, 5, 8, 9]. The high risk group includes positive resection margins (R1 or R2), high-grade tumors, multiple tumors, or extremely large tumors, with some studies defining a size of more than 10 cm [5, 8]. Owing to the high risk of radiation ileitis, radiotherapy is generally avoided in cases of mesenteric liposarcoma [11]. The use of adjuvant chemotherapy is still very limited and has no clear indications [1, 2, 7, 10, 11]. Although some studies mentioned ifosfamide, in high doses, for recurrent dedifferentiated liposarcoma, the local recurrence rate was high, approaching 40% [8]. Further studies also reported a high local recurrence rate after the application of adjuvant chemotherapy (cyclophosphamide, vincristine, adriamycin, and dacarbazine) to patients with small intestinal mesenteric liposarcomas, with five out of nine patients developing local recurrence [5]. In the present case, complete R0 resection was achieved and no high-risk features were identified, so adjuvant therapy was not indicated. The patient was therefore managed with close surveillance after multidisciplinary team discussion.

## Conclusion

Primary myxoid liposarcoma of the mesocolon is a high grade malignant tumor that has a high rate of metastases and recurrence. Surgical treatment with wide local excision to R0 margins remains the gold standard of management. Controversy arises over whether neoadjuvant and/or adjuvant treatment is needed. Although treatment protocols are still missing due to lack of sufficient reported cases, adjuvant radiotherapy is highly recommended for patients with high risk factors.

## Data Availability

Data sharing is not applicable to this article as no datasets were generated or analyzed during the current study, but details from the clinical records are available from the corresponding author on reasonable request.

## Abbreviations

AKI: Acute kidney injury
CA 19.9: Cancer antigen 19.9
CEA: Carcinoembryonic antigen
CD-99: Cluster of differentiation-99
CT: Computed tomography
DRE: Digital rectal examination
EMA: Epithelial membrane antigen
GIST: Gastrointestinal stromal tumor
Hb: Hemoglobin
HPFs: High-power fields
HU: Hounsfield Units
IHD: Ischemic heart disease
MRI: Magnetic resonance imaging
PT: Pathologic tumor stage
RC: Round cell
R0, R1, R2: Resection margins
TP53: Tumor protein p53
WD: Well differentiated
WHO: World Health Organization
